# Antibodies to Combat Fungal Infections: Development Strategies and Progress

**DOI:** 10.3390/microorganisms11030671

**Published:** 2023-03-06

**Authors:** Ali A. Rabaan, Amal H. Alfaraj, Amer Alshengeti, Abdulsalam Alawfi, Sara Alwarthan, Mashael Alhajri, Amal H. Al-Najjar, Mona A. Al Fares, Mustafa A. Najim, Souad A. Almuthree, Sultan T. AlShurbaji, Fadwa S. Alofi, Bashayer M. AlShehail, Buthina AlYuosof, Ahlam Alynbiawi, Suha A. Alzayer, Nawal Al Kaabi, Wesam A. Abduljabbar, Zakiyah A. Bukhary, Ahmed S. Bueid

**Affiliations:** 1 Molecular Diagnostic Laboratory, Johns Hopkins Aramco Healthcare, Dhahran 31311, Saudi Arabia; 2 College of Medicine, Alfaisal University, Riyadh 11533, Saudi Arabia; 3 Department of Public Health and Nutrition, The University of Haripur, Haripur 22610, Pakistan; 4 Pediatric Department, Abqaiq General Hospital, First Eastern Health Cluster, Abqaiq 33261, Saudi Arabia; 5 Department of Pediatrics, College of Medicine, Taibah University, Al-Madinah 41491, Saudi Arabia; 6 Department of Infection Prevention and Control, Prince Mohammad Bin Abdulaziz Hospital, National Guard Health Affairs, Al-Madinah 41491, Saudi Arabia; 7 Department of Internal Medicine, College of Medicine, Imam Abdulrahman Bin Faisal University, Dammam 34212, Saudi Arabia; 8 Drug & Poison Information Center, Pharmacy Department, Security Forces Hospital Program, Riyadh 11481, Saudi Arabia; 9 Department of Internal Medicine, King Abdulaziz University Hospital, Jeddah 21589, Saudi Arabia; 10 Department of Medical Laboratories Technology, College of Applied Medical Sciences, Taibah University, Madinah 41411, Saudi Arabia; 11 Department of Infectious Disease, King Abdullah Medical City, Makkah 43442, Saudi Arabia; 12 Outpatient Pharmacy, Dr. Sulaiman Alhabib Medical Group, Diplomatic Quarter, Riyadh 91877, Saudi Arabia; 13 Department of Infectious Diseases, King Fahad Hospital, Madinah 42351, Saudi Arabia; 14 Pharmacy Practice Department, College of Clinical Pharmacy, Imam Abdulrahman Bin Faisal University, Dammam 31441, Saudi Arabia; 15 Directorate of Public Health, Dammam Network, Eastern Health Cluster, Dammam 31444, Saudi Arabia; 16 Infectious Diseases Section, Medical Specialties Department, King Fahad Medical City, Riyadh 12231, Saudi Arabia; 17 Parasitology Laboratory Department, Qatif Comprehensive Inspection Center, Qatif 31911, Saudi Arabia; 18 Department of Pediatric Infectious Disease, Sheikh Khalifa Medical City, Abu Dhabi 51900, United Arab Emirates; 19 Department of Medical Laboratory Sciences, Fakeeh College for Medical Science, Jeddah 21134, Saudi Arabia; 20 Department of Internal Medicine, King Fahad General Hospital, Jeddah 23325, Saudi Arabia; 21 Microbiology Laboratory, King Faisal General Hospital, Al-Ahsa 31982, Saudi Arabia

**Keywords:** antibodies, fungal infections, cell-mediated immunity, lymphocyte, NK cells

## Abstract

The finding that some mAbs are antifungal suggests that antibody immunity may play a key role in the defense of the host against mycotic infections. The discovery of antibodies that guard against fungi is a significant advancement because it gives rise to the possibility of developing vaccinations that trigger protective antibody immunity. These vaccines might work by inducing antibody opsonins that improve the function of non-specific (such as neutrophils, macrophages, and NK cells) and specific (such as lymphocyte) cell-mediated immunity and stop or aid in eradicating fungus infections. The ability of antibodies to defend against fungi has been demonstrated by using monoclonal antibody technology to reconsider the function of antibody immunity. The next step is to develop vaccines that induce protective antibody immunity and to comprehend the mechanisms through which antibodies mediate protective effects against fungus.

## 1. Introduction

Every environmental niche, including the air, soil, freshwater, and oceans, might provide fungi that could be separated. They play a significant role in the world’s biogeochemical cycles. Many of them are also employed for human welfare. Since a very long time ago, fungi have been employed to ferment nutrients. For instance, yeasts are used to produce food both traditionally and today, as well as to break down the rash and produce items that are helpful to the industry [1,2]. Fungi have been utilized in traditional Chinese medicine for thousands of years, and recent research has concentrated on polysaccharides, an essential part of fungi cell walls [3]. Among the various polysaccharides, fungal β-glucans play a significant role in developing cosmetics, food additives, and medicine [4]. Its positive effects on the course of numerous diseases have also been demonstrated [5]. In this way, fungi contribute significantly to the biomedical and manufacturing industries and impact human health, agriculture, and biodiversity.

Although the great majority of fungus lacks pathogenic characteristics, some species inflict life-threatening superficial illnesses on humans. Invasive infections are far more difficult to cure and tremendously influence human health, leading to a significant mortality rate, whereas superficial fungal infections are typically benign [5]. There are more than a hundred species of fungi that are regarded as primary pathogens of humans and animals [6].

Primary pathogens and opportunistic pathogens are the two broad categories into which fungal pathogens can be subdivided. The fungus can establish themselves and spread infection because of their capacity to develop at 37 °C and adapt to the environment inside the host tissues [7]. Primary (true) fungal pathogens *(Blastomyces dermatitidis*, *Coccidioides immitis*, *Paracoccidioides brasiliensis*, *Histoplasma capsulatum*, *and Penicillium marneffei*) attack healthy, immunocompetent hosts and are distributed in identifiably geographic regions [8]. Opportunistic pathogens employ weak or immune-compromised hosts to spread infection and cause considerable morbidity and mortality. Over the past 20 years, fungal infections have paradoxically gained in significance as more immunosuppressed individuals have survived due to the triumph of modern medicine.

Such patients are highly susceptible to disease by opportunistic pathogens such as *Candida species*, *C. neoformans*, *A. fumigatus*, other Aspergillus species, and zygomycetes [7]. Table 1 summarizes the list of common human pathogens.

The processes of bacterial pathogens are far better understood than those of fungal infections. Fungal infections currently make for 10% of all nosocomial infections [9].

The fungus-specific factors that are responsible for the pathogenic nature of fungi are unique to that species. The enzymes and toxins significantly influence the virulence and pathogenicity of many fungi and the byproducts they produce, for example, proteases and aspartic acid proteinase, are made by Aspergillus species. The toxins that are generated by Aspergillus species include gliotoxin and restriction. An opportunistic fungus called *Candida* spp., a typical component of human flora, can lead to dangerous infections ranging from candidemia and urinary tract infections to skin and nail infections [10].

Human fungi diseases provide a severe threat but are often ignored. Fungi’s effects on human health are a growing concern. Every year, 4.9 million people are affected by invasive or persistent fungal diseases [11]. By comparison, the financial impact is significant. For example, fungal infections are estimated to have cost the United States more than USD 7.2 billion in damages in 2017 [12].

Despite the availability of antifungal medications, invasive fungal infections are thought to be responsible for 1.5 million annual deaths, a similar number to tuberculosis [13]. The global burden of common life-threatening fungal infections data have been summarized by Gago et al. (2017) in Table 2 [11].

A patient with an invasive fungal infection may incur additional medicine and hospitalization costs of between EUR 10,000 and EUR 51,000, according to limited studies on the economic impact conducted in Europe. A lack of access to diagnostic and antifungal medications is a severe issue in lower-income nations. For instance, in sub-Saharan Africa, cryptococcal meningitis is the most common cause of mortality for HIV/AIDS patients [14]. The most effective antifungal medications to treat this infection have been in circulation since the 1970s, yet they are still unavailable in many nations.

Despite the fact that some fungi can infect healthy people and cause disease, the majority of fungal infections occur in sick persons and commonly compromise the success of cutting-edge treatments for cancer, complex surgery, autoimmune disease treatments, organ and stem cell transplantation and trauma [15]. As a result, AIDS-related illnesses such as pneumocystis and cryptococcus are common in developing nations where antiretroviral medication is not widely available [16,17]. Diagnoses of invasive candidiasis, allergic bronchopulmonary disease, and invasive aspergillosis associated with cancer and surgery are becoming more common in wealthier countries [18]. The presence of fungi in the environment is a major determinant of the epidemiology of primary fungal diseases. For example, one of Latin America’s most common systemic mycoses is paracoccidioidomycosis. The geographic distribution of several paracoccidioidomycosis species in these regions significantly limits the epidemiology of this disease [19].

Antibody-based treatments have emerged as a promising approach for treating fungal infections, particularly for those caused by difficult-to-treat species or for patients who are immunocompromised. Some of the specific logic put forth by the researchers to emphasize the superiority of antibodies over other interventions includes their specificity, durability, and adaptability. Antibody-based treatments can be designed to specifically target the fungus of interest while leaving other healthy cells and microbes unharmed. This specificity can lead to fewer side effects and less damage to the patient’s immune system.

Antibodies are naturally long-lasting molecules in the body and can provide prolonged protection against infections. In contrast, some antifungal drugs need to be administered frequently and for extended periods of time, which can lead to drug resistance. Combining antibody-based treatments with other antifungal strategies, such as antifungal drugs, can lead to a synergistic effect and enhance the efficacy of the treatment, which appears to an added advantage. Moreover, antibody-based treatments can be used for prophylaxis to prevent fungal infections in high-risk patients, such as those undergoing chemotherapy or organ transplantation. Finally, antibodies can be engineered to target multiple fungal species and strains, making them adaptable to changing pathogen populations. Scientific evidence has shown the effectiveness of antibody-based treatments for fungal infections. For example, a monoclonal antibody called Mycograb was developed to target the fungus *Candida albicans*. Clinical trials showed that Mycograb improved the survival rates of patients with invasive candidiasis compared to standard antifungal therapy alone [20]. Another example is the use of passive immunization with polyclonal antibodies against *Aspergillus fumigatus* in a mouse model. The antibodies were able to reduce the fungal burden and increase survival rates compared to control mice [21].

## 2. General Mechanism of Fungal Infection

**Figure 1:**
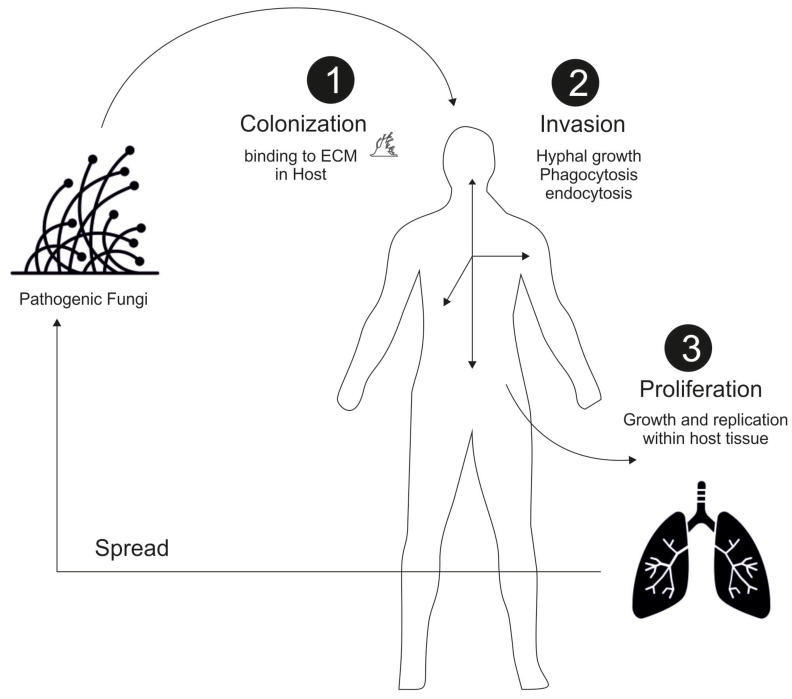
A general mechanism of the Fungal infection and pathogenesis in Humans.

When it comes to the pathogenesis of fungal infections, the nature and life cycle of fungi are crucial. As fungal infections in humans are caused by various types of fungi that can invade tissue, these infections can range from superficial skin infections to life-threatening systemic infections. The general mechanism of fungal infections in humans involves several steps, including colonization, adhesion, invasion, and proliferation of the fungi within the host tissues [22]. The first step in fungal infections is colonization, which involves the adherence of fungal cells to the host cells or extracellular matrix (Figure 1). This process is facilitated by the fungal cell walls’ components, such as adhesins and glycoproteins, which interact with host receptors. For example, Candida albicans, a common fungal pathogen, can adhere to host cells by binding to host proteins such as fibronectin and laminin [23]. Studies have shown that the adhesion of *C. albicans* to host cells is a critical step in the development of invasive candidiasis, a serious bloodstream infection. The second step in fungal infections is adhesion, which is followed by the invasion of host tissues. Fungi can penetrate host tissues through various mechanisms, such as hyphal growth, phagocytosis, and endocytosis. Once inside the host tissues, fungi can evade the host immune system by several mechanisms, including the modulation of host immune responses and the secretion of virulence factors. For example, *Aspergillus fumigatus*, a common cause of invasive aspergillosis, can produce gliotoxin, a virulence factor that suppresses host immune responses and promotes fungal growth [24]. The third step in fungal infections is proliferation, which involves the growth and replication of fungal cells within host tissues. Fungi can utilize host nutrients and evade host immune responses to promote their growth and proliferation. For example, Histoplasma capsulatum, a fungal pathogen that causes histoplasmosis, can survive and replicate within host macrophages by avoiding host immune recognition and response [25]. In addition to these steps, fungal infections can also trigger host inflammatory responses, which can contribute to tissue damage and disease progression. Such inflammatory responses triggered by fungal infections can be known to contribute to tissue damage and disease progression. Fungal cell wall components, such as β-glucans and chitin, can activate host immune cells and promote the production of proinflammatory cytokines. These responses can contribute to tissue damage and organ dysfunction. For example, studies have shown that β-glucans from *Candida albicans* can activate host immune cells and contribute to the development of systemic candidiasis [26].

## 3. Host Defense Mechanism against Fungi

So, what attributes do human fungal pathogens have? Four requirements must be met. (1) It must be capable of growing at temperatures of at least 37 °C. (2) It must be able to pass through host tissue barriers or find a way around them by penetrating air-filled spaces such as the sinuses and lungs. (3) It must be capable of absorbing and digesting parts of human tissues. (4) It must also be resilient to the human immune system [15].

The way a person’s body fights disease caused by fungi depends on their immune system. The immune system is made up of two parts: basic defenses called “innate immunity” and more complex defenses called “adaptive immunity.” These defenses are created by cells such as macrophages, monocytes, dendritic cells, PMNs, MDSCs, NK cells, alarmins, and antimicrobial peptides. The type of defense a person’s body uses against fungi depends on where the infection is located. However, the relative importance of each is primarily determined by the infection site. The innate response occurs either directly by the phagocytic mechanism, through the secretion of antifungal compounds, or through the production of cytokines and chemokines [27].

Macrophages, antigen-presenting cells (APC), take over the fungi and start different events. The macrophages of tissues work as effector cells and create cytokines and chemokines that stimulate more immune cells. Moreover, they are essential in developing granuloma [28,29]. Additionally, dendritic cells are APCs that process antigens following fungal recognition and deliver them to T cells with a further linage toward T-helper (Th) subsets, such as Th1, Th2, and Th17 cells [30]. Recent studies recognize the significance of plasmacytoid dendritic cells in aspergillosis [31]. Monocytes were classified into classical (CD14++CD16), intermediate (CD14+CD16+), and nonclassical (CD14+CD16++) subsets based on CD14 and CD16 expression [32]. Th17 differentiation is initiated by classical monocytes stimulated with IL-1 and prostaglandin E2 against *Candida albicans* [33]. Natural killer (NK) cells produce cytokines, primarily IFN-, as well as other soluble factors that regulate the activity of other immune cells [34]. NK cells harm the hyphae of both *C. albicans* [35] and *A. fumigatus* [36]. Recent research has expanded on the role of invariant NKT (iNKT) cells in cryptococcosis [37] as well as in aspergillosis [38].

The lack of Polymorphonuclear neutrophils (PMNs) shows an overall risk of invasive Candidiasis [39] and Aspergillosis [40]. They are the storehouse for several antifungal peptides, myeloperoxidase, and nucleolytic enzymes that eradicate several pathogens. Myeloid-derived suppressor cells (MDSCs) showed higher levels in people infected with either *Candida albicans* and *Aspergillus fumigatus* [41]. Β defensins, histantin, LL-37, and serprocidins are the antimicrobial peptides (AMP) which showed anti-candidacidal activity [42].

There are two types of adaptive immune responses: cell-mediated responses and humoral immune responses, and both can be elaborated in terms of the aforementioned context.

## 4. Cell-Mediated Response

Helper CD4 and cytotoxic CD8 T cells are examples of cell-mediated responses. When fungal proteins are detected, they are cleaved into small antigenic peptides, which are then assembled with MHC-I or MHC II and transported to the surface of active dendritic cells. Dendritic cells then migrate to secondary lymphoid organs, where T cells are activated. Dendritic cells then migrate to secondary lymphoid organs and activate T cells. The antigenic peptide sequence influences T cell activation. CD8 T cells contain MHC-I and MHC-II peptides, while CD4 T cells contain MHC-II related peptides.

CD4+ T lymphocytes play an important protective role in patients with human immunodeficiency virus or acquired immunodeficiency syndrome who are susceptible to fungal infections such as *Candida albicans*, *P. jirovecii*, *C. neoformans*, *and Aspergillus fumigatus* [43].

**Figure 2:**
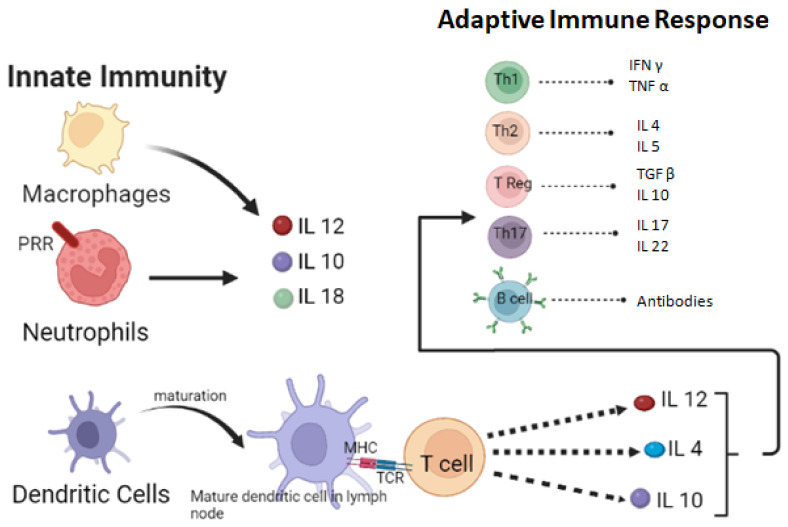
A diagrammatic representation of cellular innate response and humoral response against fungal infections.

The depletion of CD8 T cells in mice reduces survival in a cryptococcal infection model and inhibits *C. neoformans* growth in macrophages by secreting IFN g [44]. CD8 T cells become crucial and immunologically vital for suppressing fungal infections, especially when CD4 T cells are absent or perhaps have relatively low concentrations [45]. Figure 2 explains the cellular innate response and humoral response against fungal infections.

## 5. Humoral Immune Response

Glycoproteins, also known as antibodies or immunoglobulins (Igs), are critical components of the immune system. The fungal burden is reduced by these effector molecules. After binding to surface antigens, antibodies can act as opsonins, increasing the phagocytic activity of immune cells. The fungal cell wall, which is primarily composed of carbohydrate polymers and specific proteins, is the primary target structure for opsonizing antibodies. The control mechanism for fungal infections is determined by the type of fungus and the specificity of the epitope. Natural antibodies are polyreactive, have a low to medium affinity, and are frequently germ-line encoded. Natural antibodies of the IgM, IgA, and IgG classes are primarily produced by B1 cells. [46,47,48,49,50,51,52].

As we all know, a variety of antifungal antibodies protect the host against specific fungal derivatives such as glycoproteins, glycolipids or peptides, and polysaccharides. Because the majority of these components are found in the fungi’s cell wall, antifungal antibodies concentrate on its development, dynamics, and remodeling [53]. Humoral immunity has long been thought to support cellular immunity, and that the cellular immune response contributes significantly to protection.

## 6. Antibodies against Fungal Infection

Numerous studies have shown the crucial function of natural antibodies in the body’s fight against fungus infection. It has been demonstrated in multiple models that involve the transfer of normal mice serum to MT mice that they limit the proliferation of microbial fungi [54,55,56,57]. Natural antibodies have a low-to-medium affinity, are often germ-line encoded, and are polyreactive. Natural antibodies such as IgM, IgA, and IgG classes are primarily produced by B1 cells. [46,47,48,49,50,51,52]. This natural antibody’s antifungal properties include the direct reduction in germ tube development and the enhancement of candida phagocytosis through macrophage-mediated opsonization [58,59].

Studies using *Candida albicans* and *Candida neoformans*, which are two of the most well-studied and widespread human pathogenic fungi, have provided much of the current knowledge on the mechanism of AMI against fungi. In the case of *C. neoformans*, opsonization is essential for host defense because polysaccharide capsules block phagocytosis and complement is comparatively weaker than the opsonin-mediated response for a few fungal strains [60]. As *C. neoformans* can multiply intracellularly and escape phagocytosis through non-proliferative exocytosis, the efficacy of ingestion as a method of modulating this fungus depends on cellular activation and host immunity. In animal models of cryptococcosis, complement activation appears to be required for the function of specific IgMs, but it may not be required for IgGs [61,62]. According to Van Spiiel et al. (2001), Fc receptor-dependent ADCC may protect against *C. albicans* [63], while *C. neoformans* have been shown to be sensitive to NK cell antibodies [64,65]. The ability of killer toxin-mimicking antibodies to directly damage fungal cells by interacting with the killer toxin receptor serves as an example of a mediated antifungal mechanism and other antibody intervals [66]. Table 3 [67,68,69] summarizes the host PRRs (pattern recognition receptor) and their related fungal PAMPs (pathogen-associated molecular patterns).

However, in addition to T cell deficiencies, B cell deficits and hypogammaglobulinemia [70,71,72] have also been linked to cryptococcal illness [73] in patients. In addition, IgG2 deficiency has been found in healthy subjects with *C. gattii* meningitis [74], and in adults and children with HIV/AIDS with specific levels of IgG2, the polysaccharide glucuronoxylomannan (GXM) was lower than those without the disease [75,76]. In humans, IgG2 is the most common IgG subtype of the polysaccharide antigen [77].

Natural IgM are crucial for mice’s resistance to *Pneumocystis murina* and *C. neoformans* through various mechanisms. Native IgM promotes the development of Th2 cells, facilitates DC detection and the migration of fungal antigens to the draining lymph nodes, and promotes B cell class switching by recruiting macrophages to the sites of infection and improving fungal phagocytosis [78,79]. According to these results, infection with *C. neoformans* was more severe in X-linked immunodeficiency mice with much lower IgM levels [80].

Additionally, new mechanisms of antifungal antibodies have been discovered. For instance, a mAb to the mannoprotein of *Candida albicans* has been demonstrated to mediate three distinct activities: the inhibition of adhesion, suppression of germination, and direct candidacidal activity [81]. The direct effects include iron deprivation caused by the antibody.

The ability of antibodies to cell wall β-glucans to directly limit the growth of *Cryptococcus neoformans* and *C. albicans* has been shown to interfere with adhesion and cell wall remodeling [82,83]. Data demonstrating that *C. neoformans* opsonized by an antibody leaves macrophages as microcolonies that resemble biofilms, in contrast to planktonic cells that leave after complement opsonization, serve as an example of how a specific antibody can change the nature of the interaction between *C. neoformans* and macrophages [84].

Mice with B cell depletion are more susceptible to developing systemic candidiasis [85]. As demonstrated in erythemic mice challenged with *Candida albicans* at the systemic level [86], a mice model of severe combined immunodeficiency or SCID [87], and antibody-deficient animal models which lack Lyb-5 B cells [88], humoral immune responses are crucial in providing protection against systemic candidiasis.

The multiple stages of human *C. albicans* infection, from homosexuality to disease, pose a particular challenge in determining the role of AMI in disease resistance and maximizing the potential for antibody therapy in patients. Mice studies are simpler, but the results vary depending on the fungal strain, infection technique, and animal model. B-lymphocyte-depleted mice are more susceptible to filamentous *C. albicans* infection, according to previous studies [89].

Mice with B cell depletion are more susceptible to developing systemic candidiasis [85]. As demonstrated by systemic challenges with *C. albicans* in the athymic mice [86], severely combined immunodeficiency (SCID) mice [87], and antibody-deficient CBA/N mice lacking Lyb-5 B cells [88], the humoral immune response is crucial in protection against systemic candidiasis.

According to a study by Romani and colleagues, antibodies are essential for the development of memory of antifungal immunity [57]. They demonstrated that B-deficient (μMT) mice could effectively control the growth of fungal pathogens during different stages (primary and secondary) of infections by comparing their susceptibility to *Aspergillus fumigatus Candida albicans* infections via either intravenous or intratracheal routes. Thus, their findings demonstrate the significance of Th1 cells in mediating the protective response against these two fungi infections. However, μMT mice were unable to survive a subsequent *C. albicans* infection. These findings showed that even though B cells and antibodies are not necessary for Aspergillus resistance, the shield against Candida infections are facilitated by both pathways, antibody-dependent and independent [57].

Circulating healthy antibodies were found to limit the size and organization of the granulomatous lesions in the experimental paracoccidioidomycosis, a chronic granulomatous illness brought by the thermally dimorphic fungus called *Paracoccidioides brasiliensis* [90].

Critical antifungal agents should adhere to several standards [91]:

- (a) Should be a broad spectrum for a variety of fungi;
- (b) Should be fungicidal as opposed to fungistatic;
- (c) Should be against specific fungal target region;
- (d) Should have spare interference with host targets;
- (e) Should have minimal side effects or toxicities.

It was evident that finding or creating a medication that meets the aforementioned requirements is extremely difficult and necessitates discovering biological targets specific to fungus. Table 4 shows the list of some antifungal vaccines (Nami et al. (2019) with some modifications) [92].

When polyene antifungal agents interact with ergosterol in the plasma membrane, they disrupt the membrane [146], increase permeability, cause cytoplasmic leakage [147], and ultimately cause cell death [148]. According to recent research, they may also produce oxidative damage, which could help explain their fungicidal effect [149]. Amphotericin B, nystatin, natamycin (pimaricin), rimocidin, filipin, and candicin have a higher affinity for ergosterol than mammalian cholesterol, resulting in less toxicity to mammalian cells and clinically useful polyenes [150]. The advancement of polyenes is based on changes in the drug structure that convert molecular umbrella complexes into nanoparticles and polysaccharide complexes. The oral drug amphotericin B cochleate lipid crystal nanoparticles were developed and demonstrated in vivo activity [151]. Various PRRs and their counterpart PAMPs in fungi are enumerated in Table 4.

Viamet Pharmaceuticals’ azole enhancements are based on chemical changes that can modulate the metal-binding groups of azole compounds, reducing interactions with cytochrome P450s and the potential for drug–drug interactions [152]. Triazoles were shown to be more potent than itraconazole in vitro against *Candida albicans*, *Trichosporon beigelli*, *Candida parapsilosis*, *Aspergillus fumigatus*, *Candida glabrata*, and *Candida neoformans* [153].

There are three new compounds which are currently in phase II clinical trials with reference IDs: NCT02267356; NCT02267382; and VT-1129. These have been designed to combat vaginal candidiasis and onychomycosis. VT-1129 was created to treat cryptococcosis, and a related compound, VT-1598, is potent and effective against endemic mycoses and cryptococcus [153].

IPC synthase is targeted by drugs such as aureobasidin A, caspofungin, fosfomycin, 21-hydroxylstrin, galbonolide, 21-hydroxygalbonolide, and profungin A. Sphingolipids are important membrane components of fungi and mammals, and are mostly found in the fungal cytoplasmic membrane’s outer layer [154]. Serine palmitoyltransferase is an enzyme that catalyzes the condensation of serine with fatty acyl coenzyme A (acyl-CoA, usually palmitoyl-CoA) to produce the long-chain base keto-Dihydrosphingosine. This is the first and most important step in sphingolipid biosynthesis. The first fungal-specific sphingolipid biosynthetic enzyme is inositol phosphoramide (IPC) synthase [155]. It catalyzes the transfer of phosphatidylinositol from glycophosphatidylinositol to the C1 hydroxyl of ceramide to produce inositol phosphoramide.

According to Monk and Perlin [156], omeprazole, which blocks the stomach K^+^ H^+^-ATPase, can prevent the growth of *Candida albicans*. They also showed a correlation between the inhibition of the organism’s H^+^-ATPase and the inhibition of the growth of *Candida albicans*.

The first echinocandin antifungal agent licensed for use in clinical settings was caspofungin. Micafungin and anidulafungin were shortly introduced after it. These substances prevent the synthesis of -1,3-Dglucan, a crucial and essential component of the cell walls of numerous fungus, including *Candida*spp. [157,158]. It has been demonstrated that mulundocandin and deoxymulundocandin are effective against *A. niger* and *C. albicans* [159].

Rezafungin is an anidulafungin structural analogue with in vivo activity against Candida and Aspergillus species. Piperazine pyridazinones have antifungal activity against Aspergillus and Candida species in vitro [160].

Cell viability depends on chitin, which is a linear homopolymer of N-acetylglucosamine (GlcNAc, 1–4 linked). A series of membrane-localized chitin synthases (Chs1, 2, and 3) were characterized and found in *Candida albicans and Saccharomyces cerevisiae*, and Chs8 was further observed in *Candida albicans*, which synthesizes chitin and remains crucial for survival [161].

By inhibiting fungal chitin and glucan synthases, arthrichitin has a broad spectrum of activity against Candida, Trichophyton, and many plant pathogens but shows more significant activity against chitin synthase [162].

The alkaloid inhibits *C. albicans* growth by stabilizing the cleavage complex formed by *C. albicans* topoisomerase [163]. Further, it was found that the activity of topoisomerase I from *C. albicans* to eupolauridine is better than that homo sapiens enzyme [164].

No Aspergillus-licensed vaccinations are available currently to protect people from contracting aspergillosis [165]. Since protein carriers considerably enhance specific antibody responses and carbohydrate antigens are not very immunogenic, they protect *A. fumigatus* and *C. albicans* [82].

Several studies also show that mice can be immunized with the suitable fungal antigens to produce protective antifungal antibody responses. As a result, immunization with a liposomal–mannan admixture provided *C. albicans* resistance dependent on antibodies. In addition, high-titer antibodies to test antigens, including β-mannan and fructose bisphosphate aldolase (EC 4.1. 2.13), methyltetrahydropteroyltriglutamate (EC 2.1.1.14), and mycelial wall protein-1, were also demonstrated in glycoprotein-based synthetic vaccines which were primarily peptide epitopes with β-mannan. As a result of the DC immunization strategy, these antibodies also provided protection against experimental disseminated candidiasis [113].

Mucosal protective IgG and IgA antibodies against *Candida albicans* Sap2t are produced after an intravaginal vaccination with the secreted aspartic protease (Sap2t) family. Mice were protected from vaginal candidiasis by passive infusion of these or anti-Sap2t IgM and IgG monoclonal antibodies [95].

## 7. Future Prospects

Antibody-based therapies for fungal infections are an active area of research with several future perspectives and potential benefits. Researchers are developing new antibodies against various fungal pathogens, which can be used for the treatment and prevention of infections. Antibody-based therapies can be combined with other antifungal drugs to increase efficacy and reduce the likelihood of developing resistance. Advances in technologies such as genetic engineering and synthetic biology may enable the development of more effective and specific antibodies. Antibody-based therapies can be tailored to the individual patient’s needs, based on the specific fungal strain causing the infection and the patient’s immune status. However, there are also limitations and challenges associated with antibody-based therapies. The high cost of these therapies may limit their accessibility to patients who cannot afford them. Although antibodies are generally well tolerated, they can still cause adverse effects in some patients. As with all antimicrobial agents, there is a risk of developing a resistance to antibody-based therapies. Currently, there are only a few approved antibody-based therapies for fungal infections, and they may not be effective against all fungal pathogens. In terms of benefits to future humans, antibody-based therapies can improve the outcomes of fungal infections, especially for patients who are immunocompromised. They are highly specific, which can reduce the incidence of side effects compared to broad-spectrum antifungal drugs. Antibody-based therapies can also be used for prophylaxis to prevent fungal infections in high-risk patients, such as those undergoing chemotherapy or organ transplantation. With continued research to address the challenges and limitations and to develop new antibodies and technologies, antibody-based therapies offer a promising approach for treating fungal infections.

## 8. Conclusions

There is no doubt that the threat posed by FIs will continue to grow globally, but a number of challenges (such as the emergence of resistance) must be faced. At various levels, quick and inventive action is required.

By developing and improving novel therapeutic approaches, antifungal susceptibility testing for the identification of antifungal defiance, rapid diagnostics, enhancing public health capabilities by raising awareness of FIs at social and governmental levels, and conducting research on understanding fungal infection, the burden of fungus infections could be significantly reduced. The findings indicated that the primary determinant of the function of antibodies in the defense against fungi is the number of protective antibodies.

## Figures and Tables

**Table 1:** Common Human Pathogenic Fungi.

Fungal Pathogen	Disease	Organs Affected
Aspergillus	Aspergillosis	Usually occurs in people with lung diseases or weakened immune systems.
Candida	Candidiasis	Commonly affects the mouth and throat, vagina, or the bloodstream.
Cryptococcus	Cryptococcosis	Infect the lungs and/or central nervous system (cryptococcal meningitis).
Pneumocystis	Pneumocystis pneumonia	Infect mainly lungs when the host immune system becomes compromised.
Mucormycetes	Aggressive infections	Organs such the brain, spleen, heart, and skin by spreading locally and through the circulation.
Histoplasma	Histoplasmosis	Infect the lungs and/or central nervous system (cryptococcal meningitis).

**Table 2:** Load of common life-threatening fungal infections.

Fungal Infection	Number Affected	Case Fatality Rate	Estimated Deaths
Cryptococcal meningitis	223,000 in AIDS	15–20% USA >50% developing world	180,000 in AIDS
Pneumocystis pneumonia	>400,000 in AIDS >100,000 in non-AIDS	~15% in AIDS with best treatment ~50% in non-AIDS	>200,000 in AIDS >50,000 non-AIDS
Disseminated histoplasmosis	~100,000	15–30%, if diagnosed and treated	>80,000
Invasive aspergillosis	>1,000,000	~30% mortality in leukemia in HIC	>500,000
Invasive candidiasis	>750,000	~40% mortality treated	>350,000
Chronic pulmonary aspergillosis	>3,000,000	~15–40% mortality in HIC ~15% mortality in the developed world	>450,000 in non-hospitalized populations
Severe asthma with fungal sensitization (SAFS)	>6,500,000	<1% but no good figures	350,000–489,000 asthma deaths ~50% related to SAFS
Fungal keratitis	1.0–1.4 million	Blinding > 60%	>600,000 blind eyes
Total	~13,500,000		>1,600,000

**Table 3:** PRR Recognition of Fungal PAMPs.

Host PRR	Pathogens	Fungal PAMPs
CLR Dectin-1	*Candida*spp., *A. fumigates*, *Pneumocystis*spp., *Coccidioides*spp., *Fonsecaea*spp., *T. rubrum*	β (1,3)- glucan
Dectin-2	*Candida*spp., *Malassezia*spp., *A. fumigates*, *P. brasiliensis*, *Pneumocystis*spp., *C. posadasii*, *F. pedrosoi*, *H. capsulatum*, *T. rubrum*, *C. neoformans*	α-mannan, high mannose structures
Dectin-3	*C. albicans*	α-mannan
Mincle	*C. albicans*, *A. fumigatus*, *Pneumocystis*spp., *Fonsecaea*spp., *Malassezia*spp.	α-mannose
Mcl	*C. albicans*, *B. dermatitidis*	α-mannose
DC-SIGN	*C. albicans*, *A. fumigates*, *T. rubrum*, *P. brasiliensis*	Mannose, surface carbohydrate in extracellular vesicles
MR	*C. neoformans*, *P. carinii*, *C. immitis*, *P. brasiliensis*, *H. capsulatum*, *T. rubrum*	Mannose
Mannose receptor	*C. albicans*, *C. neoformans*, *A. fumigates*, *P. brasiliensis*	Mannan (N-linked), mannoproteins, mannan, gp43
Galectin-3	*C. albicans*	β-1,2-mannosides
Scarf1/CDC36	*C. albicans*, *C. neoformans*	β (1,3)- glucan
TLR TLR2	*C. albicans*, *Alternaria*, *A. fumigatus*	Phospholipomannans, α-glucans zymosan
TRL3	*A. fumigatus*	dsRNA
TLR4	*C. albicans*, *A. fumigatus*	α-, β-glucan, and galactomannan N
TLR6	*Candida*spp.	Zymosan, unmethylated DNA with CpG motif
TLR7	*Candida*spp.	ssRNA
TLR9	*C. neoformans*, *C. albicans*, *A. fumigatus*	Zymosan, CpG-oligodeoxynucleotides
NLR NLRP3	*C. albicans*, *A. fumigatus*, *C. neoformans*, *Malassezia*spp., *P. brasiliensis*, *S. schenckii*, *H. capsulatum*	Unknown
NLRP4	*C. albicans*	Unknown
NLRP10	*C. albicans*	Unknown
NOD1	*A. fumigatus*	Unknown
NOD2	*C. parapsilosis*, *A. fumigatus*	Chitin
RLR MDA5	*C. albicans*	Unknown

**Table 4:** The list of some antifungal vaccines (Nami et al. (2019) with some modifications) [92].

Pathogen	Antigen	Vaccine Type	Underlying Immune Mechanism	Reference
Candidiasis	Als3p Als1p	Recombinant protein (NDV-3)	IgG, IL17 A, IFN-γ, and IgA1,	[93,94,95,96,97,98]
	SAP2	Recombinant protein	Protective antibodies	[95,99]
	aspartyl proteinase protein, Sap2p (Secreted fraction)	Recombinant	Antibodies	[99]
	Tet-NRG1	Recombinant and live attenuated	Elicit immunity mediated by T cells	[100,101]
	PCA-2 strain of *Candida albicans*	Live-attenuated type	Elevated activity of macrophages and polymorphonuclear leukocyte	[102]
	Surface proteins of the cell wall	Protein subunit vaccine type	Immunoglobulins- and Th17 cytokine-mediated response	[103]
	Mannose derivatives of *Candida albicans*	Protein conjugate vaccine with mannan derivatives	Immunoglobulin responses	[104]
	Laminarin (Lam) β-polysaccharides	Lam diphtheria toxoid CRM197 conjugate	Passive immunoglobulin responses	[82,83,104,105,106,107]
	*C. dubliniensis* mannan and human serum albumin	Conjugate vaccine type	Immunoglobulin (IgG, IgA)-mediated and Th1-mediated responses	[108]
	Fructose bisphosphate aldolase (Fba) (cytosolic and cell wall peptides	Subunit vaccine type	Immunoglobulin response	[109,110]
	Heat-killed *C. albicans* (HK-CA)	Recombinant vaccine type	Antibody (IgG, IgA)- Th1	[111]
	Cell surface protein named Hyr 1 from *C. albicans*.	Recombinant vaccine type (using N-terminal part)	Immunoglobulin response	[112]
	Combination of peptide epitopes and β-mannan conjugates	Subunit/conjugate vaccine type	Immunoglobulin-mediated and Th1- mediated responses	[113]
Aspergillosis	Crude culture of *Aspergillus fumigatus* filtrate	Sonicated and filtered fractions subunit-based vaccine	Th1 cells-mediated IFN-γ and IL-2 cytokine-induced response	[114]
	Asp f3	Incomplete Freund’s adjuvant	Immunoglobulin- and T cell-mediated response	[115]
	Viable conidia of *Aspergillus fumigatus*	Sonicated and filtered fractions subunit-based vaccine	Not yet Described	[116]
	*Aspergillus fumigatus* hyphal sonicate (HS)	Recombinant	Immunoglobulin-mediated and T cell-mediated responses	[117]
	Heat-killed Saccharomyces sp.	Live-attenuated vaccine types	Mostly cell-mediated immune response mediated by Th1, Th2, and Th17 cytokines	[117,118]
	Cell wall epitope (p41 of gluconate, Crfl1) from *Aspergillus fumigatus*	Subunit	MHC 2-mediated T cell-based protection and protection against lethal infection with *C. albicans* in cross-section	[119]
	Asp 16 f	Recombinant vaccine type	Th1	[120]
	Asp 3 f	Recombinant vaccine type	Th1	[117]
	Various proteins Gel1p, Mep1p, Crf1p, Sod1p, Dpp5p, RNUp, Pep1p, Cat1p, Polysaccharides: β1–3 glucan, β1–3 glucan, GM, glycolipids: GSL, LGM	Recombinant/Subunit type vaccine	Th1	[98]
Blastomycosis	Adhesin BAD1 gene	Whole organism/live attenuated vaccine	Cytotoxic T cell-mediated immunity	[121]
Paracoccidioidomycosis (PCM)	gp 43 (P10)	DNA vaccine (pcDNA3-P10)	T regulatory cells-based immunological memory	[122]
	gp 43 (P10)	Recombinant protein vaccine	Th1 cell immunity with concurrent role of IL-12 and IFN-γ cytokines	[123]
	P10- FliC fusion protein	Recombinant	Th1	[124]
	rPb27	Recombinant	Immunoglobulin-based humoral immunity	[125]
	Heat shock protein (Hsp 60)	Recombinant	Helper cell-mediated immunity	[126]
	*Mycobacterium leprae* derived HSP65	Recombinant DNA	Th1	[127]
Cryptococcosis	GXM	Conjugate/soluble antigenic fractions	Anti-GMX antibodies (active immunization)	[128]
	GalXM	Subunit/conjugate Vaccine	Immunoglobulin (IgG, IgA)-mediated immunity	[129]
	Serotype A and Matα of *C. neoformans* strain H99γ	Live attenuated	Th- and cytotoxic T cell-mediated immunity	[130]
	Mutant C. neoformans strain lacking the enzyme steryl glucosidase 1 named (Δsgl1)	Live attenuated recombinant	Th- and cytotoxic T cell-mediated immunity	[131]
	CneF (culture filtrate Ags), mannoprotein	Subunit/recombinant	Immunoglobulin (IgG, IgA)-mediated and Th1-mediated responses	[132]
	GXM	GXM–protein conjugate	High-titer IgG responses	[133]
	Laminaran	Subunit (algal βglucan based)	Passive immunity	[134]
Pneumocystis	Kexin genes	Kexin-CD40 L DNA vaccine	Elevated IgG titers	[135]
	P55 protein	Recombinant protein	Th1-Th2 responses	[136]
	gp120	Recombinant protein	Immunoglobulin (IgG, IgA)-mediated and Th1-mediated responses	[137]
Histoplasmosis	Ethylenediamine extract from cell wall in aqueous formulation	Inactivated filtrated antigen combined with soluble antigenic fractions	Nd	[138]
	Ribosomes or live yeast cells of H. capsulatum	Live attenuated vaccine type	Lymphoid cell-mediated immunity	[139]
	Histone H2B–like protein	Live attenuated and recombinant type	Immunoglobulin (IgG, IgA)-mediated and Th1-mediated responses	[140]
	HSP-60	Recombinant type vaccine	Th1	[141]
	HIS-62	Recombinant protein vaccine	Cellular immune response	[142]
	80 kDa antigen	Recombinant vaccine	Immunoglobulin (IgG, IgA)-mediated and Th1-mediated responses	[143]
	Sec31 homologue	Recombinant	T cell-mediated	[144]
	H antigen	Recombinant antigen	Th1.Th2/CD8+	[145]
